# Bio-Inspired Supramolecular Chemistry Provides Highly Concentrated Dispersions of Carbon Nanotubes in Polythiophene

**DOI:** 10.3390/ma9060438

**Published:** 2016-06-02

**Authors:** Yen-Ting Lin, Ranjodh Singh, Shiao-Wei Kuo, Fu-Hsiang Ko

**Affiliations:** 1 Department of Materials Science and Engineering, National Chiao Tung University, 1001 University Road, Hsinchu 30010, Taiwan; beck.lin26@gmail.com (Y.-T.L.); chemrjd@gmail.com (R.S.); 2 Department of Materials and Optoelectronic Science, National Sun Yat-Sen University, No. 70, Lienhai Road, Kaohsiung 80424, Taiwan

**Keywords:** *π*-*s*tacking, noncovalent interaction, carbon nanotubes dispersion, hydrogen bond interaction

## Abstract

In this paper we report the first observation, through X-ray diffraction, of noncovalent uracil–uracil (U–U) dimeric *π*-stacking interactions in carbon nanotube (CNT)–based supramolecular assemblies. The directionally oriented morphology determined using atomic force microscopy revealed highly organized behavior through *π*-*s*tacking of U moieties in a U-functionalized CNT derivative (CNT–U). We developed a dispersion system to investigate the bio-inspired interactions between an adenine (A)-terminated poly(3-adeninehexyl thiophene) (PAT) and CNT–U. These hybrid CNT–U/PAT materials interacted through *π*-stacking and multiple hydrogen bonding between the U moieties of CNT–U and the A moieties of PAT. Most importantly, the U···A multiple hydrogen bonding interactions between CNT–U and PAT enhanced the dispersion of CNT–U in a high-polarity solvent (DMSO). The morphology of these hybrids, determined using transmission electron microscopy, featured grape-like PAT bundles wrapped around the CNT–U surface; this tight connection was responsible for the enhanced dispersion of CNT–U in DMSO.

## 1. Introduction

The manipulation of carbon nanotubes (CNTs) is hampered by their poor solubility in general solvents. Chemical functionalization and surface reactions of CNT sidewalls can partially alleviate this issue [1,2]. Modifying CNT surfaces by appending arenes or strong hydrogen bonding groups can enhance the material properties of CNTs and expand their applications [3]. Some of the methods for functional stabilization of CNTs (using various polymers, surfactant adsorption, or polymer grafting reactions are irreversible [4]. There are, however, versatile surface modification techniques that can maintain CNT structures [5,6]. For example, the incorporation of nucleobases into synthetic amino-grafted polymers can result in noncovalent assembly phenomena that correspond exactly with those observed after nucleobase-functionalization of small molecules. This supramolecular approach should not disrupt the structures of CNTs nor affect their unique mechanical properties. Bio-inspired noncovalent bonding of CNTs with both single- and double-stranded DNA has several promising applications [7].

A main challenge when applying noncovalent bonding interactions in CNT systems is determining where to position the complimentary moieties in the conjugated polymers. The interaction of conjugated polymers with respective to the aromatic surfaces of the CNTs can be optimized favorably through conjugated *π*-*π* interactions and highly oriented multiple hydrogen bonding interactions [8,9,10]. *π*-stacking plays an important role affecting charge transport through the extended aromatic *π*-systems of DNA, oligonucleotides, and polyaromatic systems [11,12]. The *π*-stacking of consecutive nucleic acid bases in DNA and RNA and their inter-chain hydrogen bonding control both the stability and functionality of these polymeric molecules [13]. Therefore, nucleic acids have great potential for use as stabilizers for the dispersion of CNTs. Uracil (U) is particularly interesting because it the smallest nucleic acid base. It has received great attention in recent years, with molecular simulations revealing the extent to which attractive forces can stabilize stacked U–U dimers through a combination of noncovalent electrostatic, delocalization, or dispersion interactions [14,15,16]. Multiple hydrogen bonding and *π*-stacking in nucleic complexes are important aspects of stabilizing U–U dimer species. In contrast to thymine (T), the behavior of U–U dimers has not been investigated experimentally in a systematic manner. Notably, there were preferentially incorporated discrepancies of chemical reactivity for the methyl group once grafted onto pyrimidine [17]. As to the noncovalent bonding of U or T with respect to CNTs, the overall properties of the conjugate molecules are similar due to the only difference being the methyl group for U and T. Nevertheless, U can form strong base pairs with several other bases–adenine (A), cytosine, and guanine in DNA, while T assembles only with A. Therefore, examining the properties of CNTs functionalized with U bases is interesting because of the several types of interactions available to form stabilized U complexes, as well as U–U dimers through intermolecular hydrogen bonds and *π*-stacking interactions [18].

In this study, we functionalized CNTs with U units (forming a CNT–U derivative) and grafted A units onto poly(3-hexylthiophene) (forming a so-called PAT derivative), and then examined the physical properties of these molecules. Most importantly, we investigated the degrees of dispersion and stabilization of CNT–U in the presence of PAT in a high-polarity solvent. Accordingly, we evaluated the contributions of the noncovalent interactions, multiple hydrogen bonding, and *π*-stacking on the enhanced dispersion of CNT–U.

## 2. Experimental

### 2.1. Materials

**Scheme 1:**
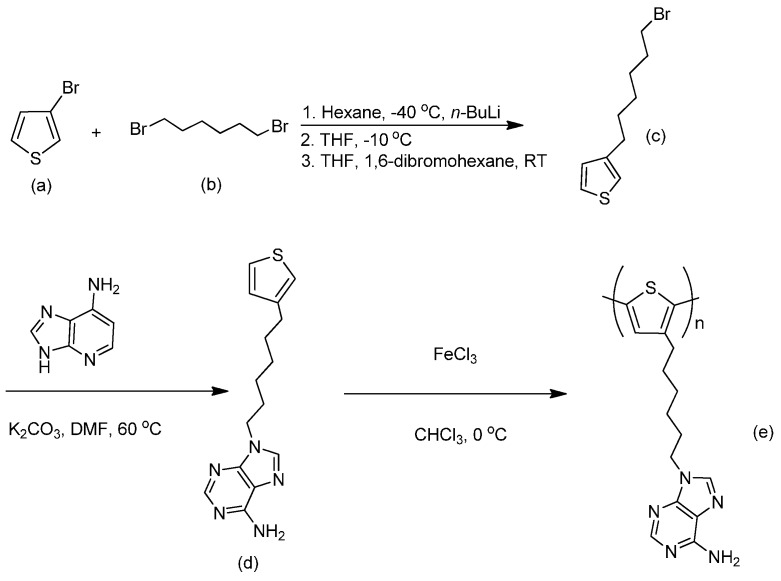
Synthesis of poly(3-adeninehexyl thiophene) (PAT).

Bromoacetic acid methyl ester (≥98%), U (≥99%), and 1,3-propanediamine (≥98%) were purchased from Alfa Aesar (Heysham, UK). 3-bromothiophene (≥97%), 1,6-dibromohexane (96%), iron(III) chloride (FeCl_3_, anhydrous, ≥98%), and chloroform (CHCl_3_, anhydrous, ≥99%) were purchased from Sigma–Aldrich (Steinheim, Germany). Dimethylformamide (DMF) (Alfa Aesar, Heysham, UK) was dried and distilled over CaH_2_ (Sigma Aldrich, Steinheim, Germany) under N_2_ (ChiahLung, Hsinchu, Taiwan). Tetrahydrofuran (THF) (Alfa Aesar, Heysham, UK) was dried under reflux for 24 h over CaH_2_ and then distilled under N_2_. All other commercially available reagents and anhydrous solvents were used without further purification. CNTs were synthesized through thermal chemical vapor deposition. The deposited products were analyzed using transmission electron microscopy (TEM; JEOL JEM-1200CXII) (JEOL, Pleasanton, CA, USA); only multiwalled CNTs (MWCNTs) were used in this study. The CNTs had diameters of 20–30 nm and lengths of greater than 1 mm. They were ground through mechanical ball milling [19] and then purified in a mixture of H_2_SO_4_ and HNO_3_ (Sigma Aldrich, Steinheim, Germany) (3:1, v/v; diluted with DI water: 60:40, v/v) at 80 °C for 4 h [20]. The synthesis (Scheme 1) of PAT has been described previously [21]. The synthesis details please see surportting information.

#### 2.1.1. Synthesis of U–NH_2_

**Scheme 2:**
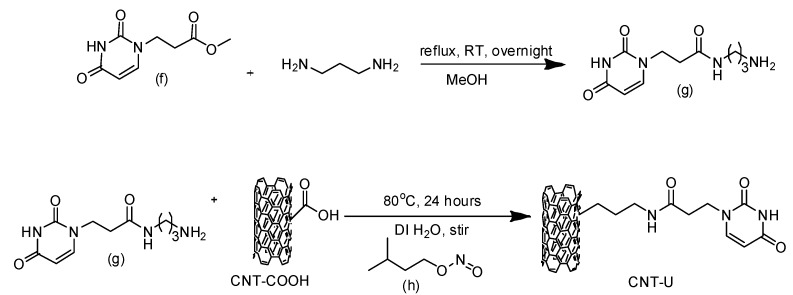
Synthesis of U-functionalized CNT derivative (CNT–U). U, uracil; CNT, carbon nanotubes.

In Scheme 2, precursor (f) was obtained from a typical S_N_2 reaction, reacted 167 g of Bromoacetic acid methyl ester (Sigma Aldrich, Steinheim, Germany) with 112 g of uracil (Sigma Aldrich, Steinheim, Germany) in 200 mL THF (Alfa Aesar, Heysham, UK). 160 g of 1,3-propanediamine (Sigma Aldrich, Steinheim, Germany) and 150 mL of MeOH (Alfa Aesar, Heysham, UK) were placed in a 250 mL double-neck bottle (DongGuang, Hsinchu, Taiwan), into which 10.5 g of the compound f was injected by syringe (DongGuang, Hsinchu, Taiwan). Ultrasonication was applied to dissolve the mixture. The mixture was heated at 80 °C overnight and then the temperature was lowered to 40 °C for 48 h duration. The mixture was concentrated through rotary evaporation; 1,3-propanediamine was distilled out under reduced pressure. The residue was washed with ether, subjected to ultrasonic treatment under ether, and then stored in a fridge (LG Corporation, FuShan, Korea). The lower phase was collected and any remaining ether was evaporated in a high-vacuum system. The product U–NH_2_, (g) was dried in a freeze dryer (Kingmech, Taipei, Taiwan) for 48 h to give the target compound (yield: 82%, 27.5 g). The structure was confirmmed by ^1^H NMR (D_2_O, 300 MHz; please see Figure S1): *δ* = 1.50 (t, 2H), 1.60 (d, 2H), 2.50 (d, 2H), 2.68 (s, 2H), 3.05 (s, 2H), 3.53 (d, 1H), 3.86 (d, 2H), 4.70 (s, 1H), 5.59 (s, 1H), 7.25 (s, 1H).

#### 2.1.2. Preparation of U–Grafted Carbon Nanotubes (CNT–U)

A mixture of 60 mg CNT–COOH (labmade) and the 4.8 g of U–NH_2_, (g) in 100 mL Deionized water (Merck Ltd., Taipei, Taiwan) were rigorously stirred and ultrasonicated in a 250 mL double-neck bottle for 1 h. Next, 2.34 g Isopentylnitrile (h) (Sigma Aldrich, Steinheim, Germany) was then slowly injected into the mixture via syringe. The resulting mixture was heated under reflux at 80 °C with vigorous stirring for 24 h. The crude product was subjected to PTFE filtration (Poly(tetrafluoroethylene) membrane, Merck KGaA, Darmstadt, Germany) and centrifugation (3000 rpm, 5 min) to produce CNT–U. Fourier transform infrared (FTIR) spectroscopy (Figure S2): 3200–3400 cm^−1^ (NH stretching), 1650 (C=O) cm^−1^ [22,23]; Raman spectroscopy (Figure S3): 1580 cm^−1^ (intense G band; Raman-allowed phonon high-frequency mode), 1350 cm^−1^ (disordered-induced D band, presumably originating from defects in the curved graphene sheets and tube ends [24,25]).

#### 2.1.3. Preparation of CNT–U/PAT Nanocomposites

CNT–COOH and CNT–U were blended with PAT in dimethyl sulfoxide (DMSO) (Alfa Aesar, Heysham, UK) at a CNT–COOH/PAT ratio (mg/mg) of 5:10 and at CNT–U/PAT ratios of 3.8:10, 6.6:10, 8:10, and 10:10. The complexes were heated at 80 °C for 24 h. After cooling, the nanocomposite prepared at a ratio (mg/mg) of 8:10 was selected for characterization.

### 2.2. Characterization

^1^H and ^13^C NMR spectra were recorded at 300 MHz and 75 MHz, respectively, using a Bruker DPX-300S spectrometer (Bruker, Billerica, MA, USA) equipped with a 9.395-T Bruker magnet. The samples (*ca*. 7 mg for ^1^H NMR; *ca*. 25 mg for ^13^C NMR) were dissolved in a deuterated solvent (Alfa Aesar, Heysham, UK) and analyzed at room temperature. Differential scanning calorimetry (DSC) was performed using a DuPont 910 DSC-9000 controller (DuPont, Wilmington, NC, USA) operated under a dry N_2_ atmosphere. The samples were weighed (*ca.* 5–10 mg) and sealed in an aluminum pan (DuPont, Wilmington, NC, USA), and then heated from 0 to 100 °C at a scan rate of 20 °C/min. The glass transition temperature was taken as the midpoint of the heat capacity transition between the upper and lower points of the deviation from the extrapolated glass and liquid lines. Thermogravimetric analysis (TGA) was performed using a TA Instruments TGA 2050 analyzer (TA Instruments, West Sussex, UK) operated at a heating rate of 20 °C/min from room temperature to 800 °C under a continuous flow of N_2_. FTIR spectra were recorded using a Thermo Nicolet Avatar 320 FTIR spectrometer (Thermo electronic, Madison, WI, USA); 32 scans were collected at a spectral resolution of 1 cm^−1^. Conventional KBr pellet (Sigma Aldrich, Steinheim, Germany) and Si substrate (Summit-Tech, Hsinchu, Taiwan) methods were employed. The sample was dissolved in DMSO and then cast onto a KBr pellet and Si substrate. UV–Vis and photoluminescence (PL) spectra were recorded using an HP 8453 diode-array spectrophotometer (Hewlett Packard, Palo Alto, CA, USA) and a Hitachi F-4500 luminescence spectrometer (Hitachi, Tokyo, Japan), respectively. Absolute quantum yields were measured using an integrating sphere (Horiba Jobin Yvon, Edison, NJ, USA) and a Horiba Jobin Yvon FluoroLog-3 PL spectrometer (Horiba Jobin Yvon, Edison, NJ, USA). The samples were sputtered with Pt prior to morphological observation using a field-emission scanning electron microscope (FESEM, Hitachi S-4700) (Hitachi, Tokyo, Japan) operated at an accelerating voltage of 15 kV. X-ray diffraction (XRD) spectra of various powders were obtained using a Rigaku D/max-2500 X-ray diffractometer (Rigaku, Woodlands, TX, USA). The radiation source was Ni-filtered Cu K*α* radiation (Rigaku, Woodlands, TX, USA) at a wavelength of 0.154 nm. The voltage and current were set at 30 kV and 20 mA, respectively. The sample was mounted on a circular sample holder and the data were collected using a proportional counter detector over the 2*θ* range from 2° to 40° at a rate of 5° min^−1^. Bragg’s law (n*λ* = 2d × sin*θ*) was used to compute the *d*-spacing corresponding to the complementary behavior. X-ray photoelectron spectrometry (XPS) was conducted by using a commercial system (Thermo VG Scientific Escalab 220i–XL) (Thermo Scientific, Waltham, MA, USA)with a monochromatic Al K*α* source and a hemispherical electron energy analyzer and 32 channel detectors. The power applied to the X-ray anode was decreased to 90 W to avoid sample degradation. The instrument work function was calibrated to give a binding energy of 83.96 eV for the Au 4f_7/2_ line of metallic gold. The samples were kept at room temperature for a week prior to analyses; the pressure in the analysis chamber was less than 5 × 10^−10^ torr. All spectra were recorded at a 90° take-off angle. TEM images were recorded using a JEOL instrument instrument operated at 120 kV; samples were dip-coated from a freshly made solution onto a carbon-coated copper grid (Sigma Aldrich, Steinheim, Germany). Atomic force microscopy (AFM) was performed using a NanoScope IIIa atomic force microscope (Digital Instrument, EnviroScope) (Bruker, Billerica, MA, USA) at room temperature.

## 3. Results and Discussion

### 3.1. Spectroscopic and Thermal Properties of CNT–U

**Figure 1:**
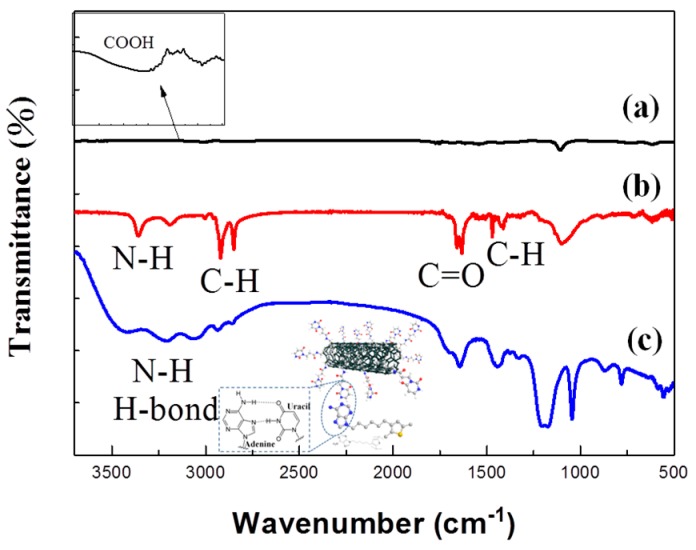
Fourier transform infrared (FTIR) spectra of (**a**) CNT–COOH; (**b**) CNT–U; and (**c**) the CNT–U/PAT (8:10) hybrid, recorded at room temperature on a Si substrate.

**Figure 2:**
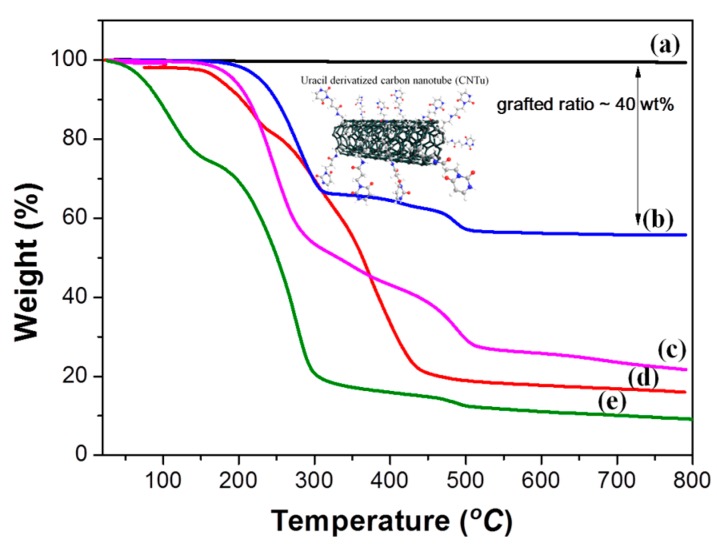
Thermogravimetric analysis (TGA) thermograms of (**a**) CNT–COOH; (**b**) U–NH_2_; (**c**) CNT–U; (**d**) the CNT–U/PAT (8:10) blend; and (**e**) PAT.

First, we evaluated the properties of commercially available CNTs and our homemade MWCNTs by recording their UV–Vis spectra at various temperatures in DMSO (Figure S4). We observed a greater extent of variation in the absorption signals of the CNTs with respect to the temperature, suggesting lower purity, than we did for our home-made CNTs. In other words, our homemade CNTs had more uniform thicknesses and more homogeneous lengths. Thus, we selected our homemade CNTs for subsequent studies of their dispersion ability through bio-inspired supramolecular interactions. Figure 1 displays FTIR spectra of CNT–COOH, CNT–U, and CNT–U/PAT blends, recorded at room temperature. In the spectrum of CNT–COOH (a), an extremely week signal appeared near 3300 cm^−1^ for the COOH units. For CNT–U, intense signals appeared for NH and C=O stretching at 3200–3400 cm^−1^ and 1650 cm^−1^, respectively, which is consistent with its successful synthesis from the reaction of U derivative and CNT–COOH (Scheme 2). The FTIR spectrum of the CNT–U/PAT hybrid is consistent with the existence of complexation. The signal for NH stretching had broadened, suggesting that the U groups of CNT–U were interacting with the A group of PAT through orientated hydrogen bonds. Thermal analysis can be an effective approach for estimating the degrees of immobilization of molecules upon CNT surfaces [26]. The ratio of G-band/D-band in the Raman spectra for CNT–COOH and CNT–U is 1.74 and 1.22, respectively, as CNT–U has a higher D band. This was due to the disruption of SP2 bonds of the carbon as C=C had been grafted with functional groups (U moiety). In general, the properties of a modified CNT surface depend on how efficiently the surface has been modified [27]. Figure 2 presents TGA thermograms, recorded under a N_2_ atmosphere, of CNT–COOH, U–NH_2_, CNT–U, the CNT–U/PAT (8:10) blend, and PAT. The thermogram of CNT–COOH reveals its excellent thermal resistance up to 800 °C. The thermogram of U–NH_2_ reveals significant loss of mass at temperatures from 175 to 450 °C, presumably because of decomposition of the C=O group and U moiety. The 40 wt% decomposition displayed by CNT–U at 300 °C suggests a high degree of grafting of U moieties on the MWCNT surface. The 40 wt% loss at a temperature of 300 °C in the TGA trace of the CNT–U/PAT hybrid was again due to thermal decay of the U units of CNT–U; the subsequent 40 wt% loss at 500 °C was due to thermal decay of the attached PAT through intermolecular hydrogen bonding of U···A between CNT–U and PAT. The loss in mass of approximately 40% corresponds to one U derivative group per 28 carbon atoms determined using the formula [23]:

(1) $$\mathrm{Grafting}\;\mathrm{ratio}=\frac{\left(100-\mathrm{wt}\%\;\mathrm{loss})/M(Carbon\right)}{wt\%\;loss/M\;(U-NH2)}$$

### 3.2. Effect of Grafting Uracil onto CNTs

**Figure 3:**
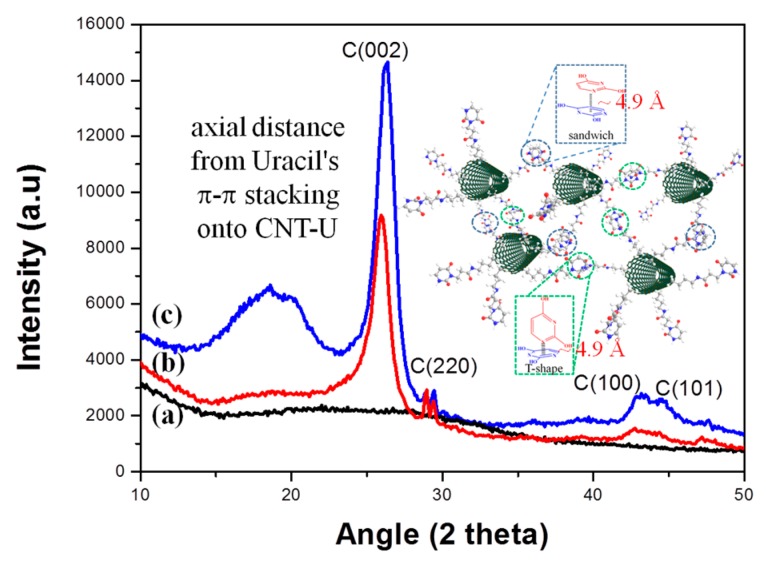
Wide angle X-ray diffraction patterns of (**a**) U–NH_2_; (**b**) CNT–COOH; and (**c**) CNT–U.

The applications of CNTs are constrained by their lack of solubility in aqueous environments, as well as their biotoxicity, due to their hydrophobic surfaces. We suspected that CNT–U might overcome these limitations. We used XRD spectroscopy to examine the difference in chemical periodicity upon grafting CNT–COOH with U moieties (Figure 3). The characteristic peaks of CNT–COOH and CNT–U appeared at values of 2*θ* of 26°, 29°, 43°, and 45°, assigned to the distinguishable (002), (220), (100), and (101) planes, respectively [24,25,28]. Interestingly, the XRD pattern of CNT–U featured a broad peak at 18°–19° that was not present for CNT–COOH. We attribute this specific XRD signal to the *d*-spacing arising from interactions among various CNT–U molecules. The resulting estimated distance between CNT–U molecules of approximately 4.9 Å corresponds to the distance in a *π*-stack [29]. Thus, we suggest that this distance is the axial distance between U moieties *π*-stacking on CNT–U; it is consistent with highly regular and extensive U modification on the CNTs. It further implies that the U molecules interacted through *π*-stacking of the U–U dimer with the hexagonal surface of the CNT–U [30]. The U–NH_2_ is extremely moisture-sensitive, displaying rapid deliquescence upon its transfer from under vacuum to the XRD holder. The presence of nucleic acid moiety was responsible for this compound having strong affinity for moisture.

**Figure 4:**
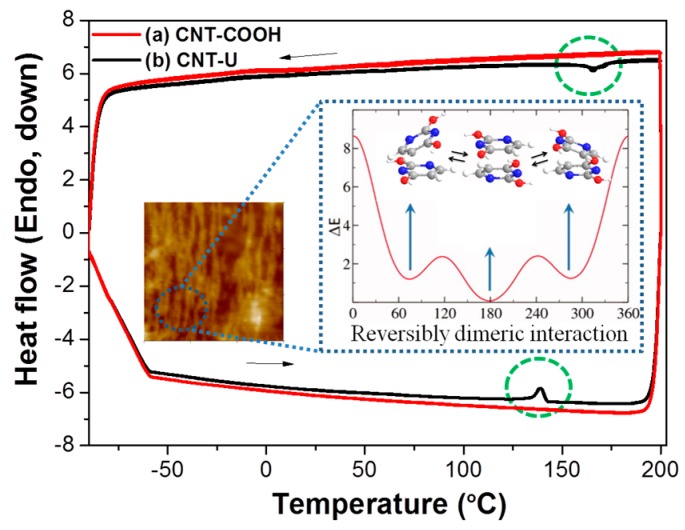
Differential scanning calorimetry profiles of (**a**) CNT–COOH and (**b**) CNT–U. Each sample was subjected to three temperature cycles ranging from −100 to 200 °C. Insets: atomic force microscopy (AFM) image and energy diagram for a reversible *π*-stack interaction.

We recorded DSC traces for CNT–COOH, U derivative, and CNT–U, scanning over the temperature range from –100 to 200 °C with three test cycles (Figure 4). The heating cycle for CNT–COOH revealed stable, thermally reversible properties upon temperature stressing, presumably because of the excellent thermal stability of this CNT–U material. For CNT–U, we observed a small heat variation at temperatures near 135 °C and 165 °C. The endothermic peak at 135 °C suggests that CNT–U acquired energy to overcome some sort of physical hindrance, possibly some sort of intramolecular interaction. Therefore, we suggest that a multiple hydrogen bond interaction and another reversible interaction were additional noncovalent intermolecular interactions displayed by CNT–U. These interactions are well-oriented *π*-stacked interactions as observed from the energy diagram of the U stacking flipping behavior. Then, multiple hydrogen bonds from the N and O atoms of the heterocycle are localized in a favorable configuration for stabilizing CNT–U matrixes. The DSC characteristics suggest that the *π*-stacking interactions of U–U dimers are reversible and controllable [31,32,33].

**Figure 5:**
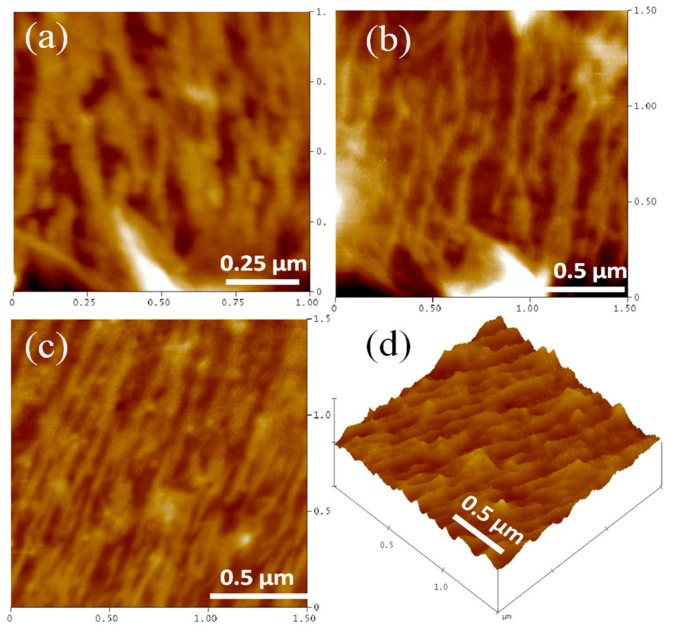
Atomic force microscope images (tapping mode: 1.5 µm × 1.5 µm) revealing the self-assembly of CNT–U through *π*-stacking of U–U dimers. (**a**) Magnified view of the CNT-U matrix’s; (**b**,**c**) different views of the CNT-U matrix; (**d**) cross-section view of the CNT-U matrix.

To observe evidence for equilibrium forces between CNT–U units, we recorded surface AFM morphological images (Figure 5). The AFM images reveal a regular and directional morphology, consistent with the behavior of the interaction force mentioned above. The directional orientation in the AFM image reveals that the orientation of the CNT–U units could be regulated by manipulating the tactility for a selected noncovalent interaction. The regular arrays of CNT–U indicates strong organizing behavior through *π*-stacking between the U moieties of the CNT–U [12,13,14,28,29,33]. These interactions are presumably the same reversible interactions inferred from the DSC traces and the well-oriented *π*-stacks inferred from the XRD analysis.

### 3.3. Supramolecular Interactions in CNT–U/PAT Dispersions: Structure and Morphology

**Figure 6:**
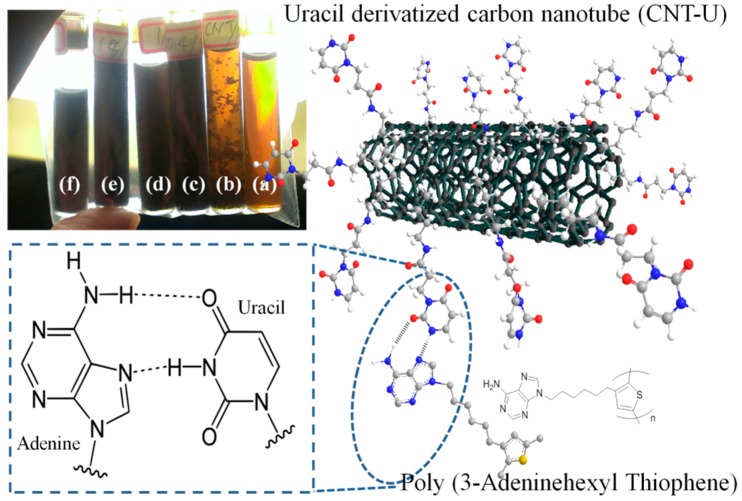
Dispersion properties of various molecules in DMSO. (**a**) PAT; (**b**) CNT–COOH; CNT–U/PAT blends at ratios (mg/mg) of (**c**) 3.8:10; (**d**) 6.6:10; (**e**) 8:10; and (**f**) 10:10. Schematic hydrogen bonding between U and A, red ball: Oxygen, blue ball: Nitrogen, gray ball: Carbon, white: Hydrogen.

Next, we examined whether reinforced CNTs could be dispersed in polar solvent systems through bio-inspired supramolecular interactions. Accordingly, we monitored the solution behavior of the polymer PAT, CNT–COOH, and CNT–U/PAT blends in DMSO (Figure 6). The control sample of PAT dissolved readily in DMSO, but CNT–COOH exhibited serious aggregation and did not disperse in the presence of PAT, consistent with no hydrogen bonding occurring between the CNT and PAT. Interestingly, the CNT–U/PAT mixtures exhibited improved dispersion properties that depended on their blend ratios. The interactions in the CNT–U/PAT hybrids presumably included *π*-stacking and multiple hydrogen bonding between the U moieties of CNT–U and the A moieties of PAT. Most importantly, the U···A multiple hydrogen bonding interactions of CNT–U and PAT enhanced the dispersion of CNT–U in DMSO, a high-polarity solvent.

**Figure 7:**
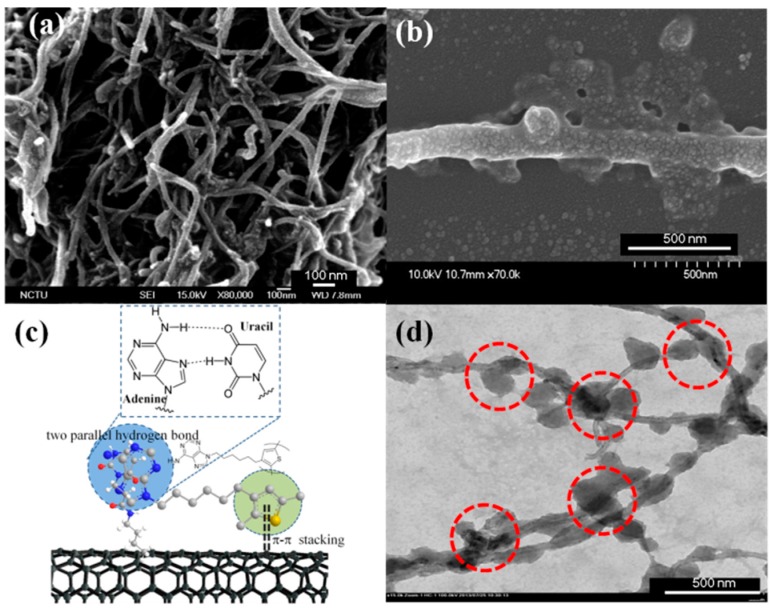
(**a**) Scanning electron microscope image of loose structure of CNT–U/PAT (8:10); (**b**) Magnified view of the SEM image of the surface in (**a**); (**c**) Structural representation of *π*-stacking and U···A multiple hydrogen bonding; (**d**) Transmission electron microscopy image of grape-like assemblies of PAT attached to CNT–U in the CNT–U/PAT (8:10) nanocomposite.

The morphology of the CNT–U/PAT hybrid contributed to its greater dispersion ability. Prior to characterizing the morphology using SEM and TEM, we removed the DMSO solvent from the CNT–U/PAT (10/10) through vacuum evaporation. The morphology of the CNT–U/PAT hybrid (Figure 7a) was that of a homogeneous dispersion with some concentrated CNT bundles. In this CNT–U/PAT hybrid material, the nanocomposites were arranged in a loose structure, presumably because of strong multiple hydrogen bonding interactions between the base units in CNT–U and PAT. In Figure 7b it appears that PAT had wrapped and interacted with CNT–U through the H-bonding forces depicted in Figure 7c. This magnified SEM image, focusing on the framed area, reveals a few nanosized PAT bundles attached to the fractured surface (despite the bundle orientation, with respect to the image plane, not being known) [29,30]. Figure 7c depicts the *π*-stacking interactions between the surface of CNT–U and the thiophene rings of PAT; the U···A multiple hydrogen bond interaction enhanced the dispersion of CNT–U in the PAT solution. To further identify the association of CNT–U and PAT, the TEM image of the hybrid material is presented in Figure 7d. We have marked various sites of interest where grape-like assemblies of PAT are attached to the CNT–U surface. Most of the CNT–U surface was covered with PAT. Hence, the enhanced dispersion of CNT–U in DMSO arose from the beneficial effect of the outer coating of PAT [34,35,36,37] Please see Figure S5.

### 3.4. Mechanism of Interaction and Energy Transfer for CNT–U and PAT

**Figure 8:**
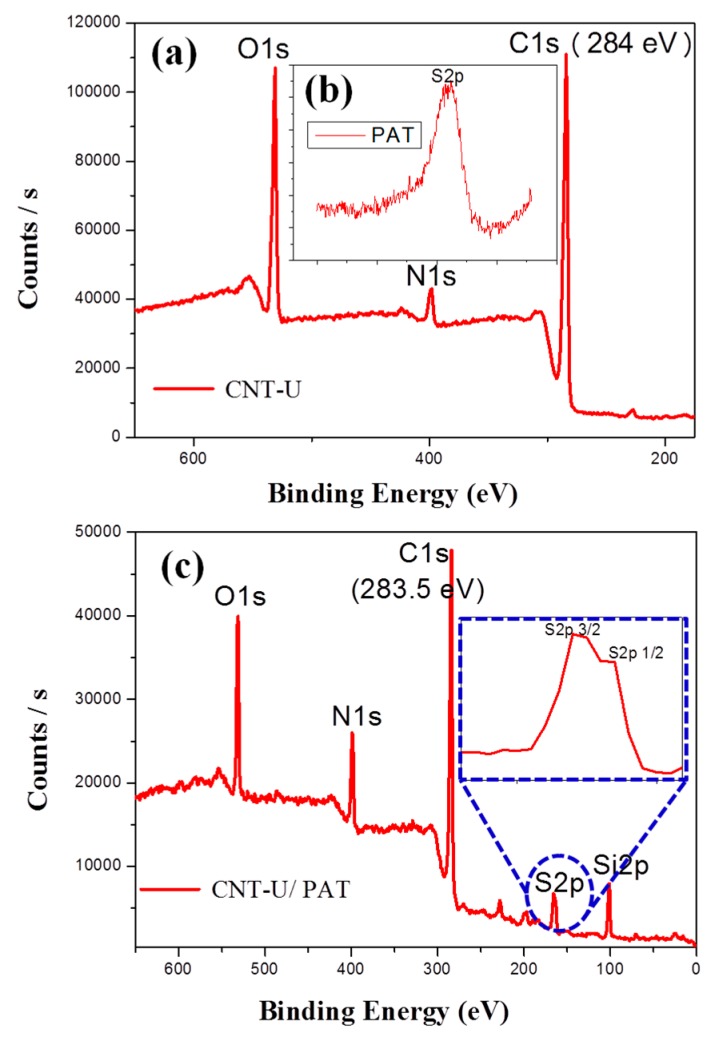
X-ray photoelectron spectrometry (XPS) spectra of (**a**) CNT–U; (**b**) PAT; and (**c**) the CNT–U/PAT blend.

**Figure 9:**
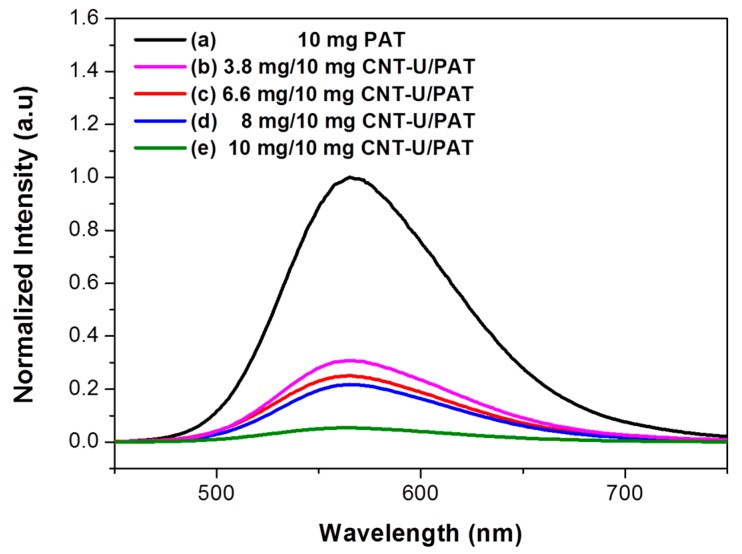
Fluorescence photometry (excitation wavelength: 396 nm) of (**a**) PAT and (**b**–**e**) CNT–U/PAT blends at ratios (mg/mg) of (**b**) 3.8:10; (**c**) 6.6:10; (**d**) 8:10; and (**e**) 10:10.

*π*-Stacking–induced charge transfer phenomena can have major effects on conjugate molecules Please see Figure S6 [11,12]. The above-mentioned association of grape-like PAT assemblies with CNT–U occurred through *π*-stacking interactions in addition to hydrogen bonding. We used XPS (Figure 8) and fluorescence spectrometry (Figure 9) to examine the effects of these interactions on the energy transfer processes of these molecules. In Figure 8a we observe various signals for the binding energies of CNT–U, assigned to O_1s_ (530 eV), N_1s_ (400 eV), and C_1s_ (284 eV) signals. We assign the binding energy at 164.5 eV for PAT in Figure 8b to its S_2p_ signal. The *π*–*π* interactions of CNT–U and PAT resulted in shifts in these binding energies (Figure 8c). Interestingly, the binding energy of the C_1s_ signal shifted from 284 to 283.5 eV, while the S_2p_ signal transformed from a singlet (164.5 eV) to a doublet (164.9 eV and 165.3 eV). The red-shift of the binding energy for the C_1s_ signal (CNT–U) and the blue-shift for the S_2p_ signal (PAT) are consistent with charge transfer occurring from PAT to CNT–U as a result of *π*-stacking. As we mentioned in our discussion regarding the reversible *π*-stacking in AFM, there were additional noncovalent intermolecular interactions displayed by XPS in Figure 8; the offset *π*-stacked geometry and *π*–*σ* attraction in a T interaction contributed to the doublet effect. The offset *π*-stacked geometry provided orbital overlap stronger than that in the T-type *π*–*σ* interaction. Therefore, the binding energy for the *π*–*π* interaction force was represented by the S_2p_ signal at 165.3 eV, while that for the *π*–*σ* attraction force appeared at 164.9 eV.

The improved dispersion of CNT–U in DMSO as a result of supramolecular interactions with PAT resulted in tunable fluorescence. To obtain Figure 9a we used an excitation wavelength of 396 nm to excite the PAT chromophore and then observed the fluorescence emission signal at 570 nm [37]. Interestingly, increasing the amount of CNT–U dispersed in DMSO led to increased quenching of the fluorescence emission (Figure 9b–e) [38,39]. Thus, the molecular fluorescence of PAT can be tuned through supramolecular interactions with CNT–U. In other words, we observed evidence for energy transfer from PAT to CNT–U [40,41,42,43].

## 4. Conclusions

We have used several experimental techniques to demonstrate the specific affinity between CNT–U and PAT. Specifically, we also found that U–U interactions stabilize CNT–U arrays, the reversible *π*-stacking helps us to recognize the biological functions and effects of nucleic acid modifications after inducing U onto CNTs. From photographic, SEM, and TEM images, we observed stable dispersions of CNT–U in PAT solution in DMSO, and that energy transfer experiments by fluorescence can expand the CNT–U steady dispersion through an energy transferring procedure. Furthermore, we report here the first observation, based on XRD and AFM analyses, of U–U dimeric *π*-stacking in this designed CNT–U dispersion system. We suspect that such dispersions of CNT–U with PAT in DMSO could be applied in the production of high-quality thin films and nanocomposite materials.

## Supplementary Materials

The supplementary materials are available online at www.mdpi.com/1996-1944/9/6/438/s1.

## Abbreviations

The following abbreviations are used in this manuscript:

MWCNTs	Multi-walled Carbon Nanotubes;
CNT–U	Uracil-functionalized CNT derivative;
CNT	Carbon Nanotube;
PAT	Adenine-terminated poly(3-adeninehexyl thiophene);
DMSO	Dimethyl sulfoxide;
U–U	Uracil–Uracil.

## References

[1] Fernandes F.M., Ruiz-Hitzky E. (2014). Assembling nanotubes and nanofibres: Cooperativeness in sepiolite–carbon nanotube materials. Carbon.

[2] Shih H.-K., Hsieh C.-C., Mohamed M.G., Zhu C.-Y., Kuo S.-W. (2016). Ternary polybenzoxazine/poss/swcnt hybrid nanocomposites stabilized through supramolecular interactions. Soft Matter..

[3] Dehghan M., Al-Mahaidi R., Sbarski I., Mohammadzadeh E. (2015). Surfactant-assisted dispersion of mwcnts in epoxy resin used in cfrp strengthening systems. J. Adhes..

[4] Primo E., Gutierrez F., Luque G., Dalmasso P., Gasnier A., Jalit Y., Moreno M., Bracamonte M., Rubio M.E., Pedano M. (2013). Comparative study of the electrochemical behavior and analytical applications of (bio) sensing platforms based on the use of multi-walled carbon nanotubes dispersed in different polymers. Anal. Chim. Acta.

[5] Morishita T., Matsushita M., Katagiri Y., Fukumori K. (2011). Effects of the composition and molecular weight of maleimide polymers on the dispersibility of carbon nanotubes in chloroform. Carbon.

[6] Erdem A., Papakonstantinou P., Murphy H., McMullan M., Karadeniz H., Sharma S. (2010). Streptavidin modified carbon nanotube based graphite electrode for label-free sequence specific DNA detection. Electroanalysis.

[7] Yang C.-C., Lin Y.-C., Wang P.-I., Liaw D.-J., Kuo S.-W. (2014). Polybenzoxazine/single-walled carbon nanotube nanocomposites stabilized through noncovalent bonding interactions. Polymer.

[8] Yang J., Yu G., Xia D., Huang F. (2014). A pillar [6] arene-based uv-responsive supra-amphiphile: Synthesis, self-assembly, and application in dispersion of multiwalled carbon nanotubes in water. Chem. Commun..

[9] Baykal B., Ibrahimova V., Er G., Bengü E., Tuncel D. (2010). Dispersion of multi-walled carbon nanotubes in an aqueous medium by water-dispersible conjugated polymer nanoparticles. Chem. Commun..

[10] Lillehei P.T., Kim J.-W., Gibbons L.J., Park C. (2009). A quantitative assessment of carbon nanotube dispersion in polymer matrices. Nanotechnology.

[11] Rathore R., Burns C.L., Abdelwahed S.A. (2004). Hopping of a single hole in hexakis [4-(1,1,2-triphenyl-ethenyl) phenyl] benzene cation radical through the hexaphenylbenzene propeller. Org. Lett..

[12] Gervasio F.L., Laio A., Parrinello M., Boero M. (2005). Charge localization in DNA fibers. Phys. Rev. Lett..

[13] Wang C.-F., Kuo S.-W., Lin C.-H., Chen H.-G., Liao C.-S., Hung P.-R. (2014). Benzoxazine as a reactive noncovalent dispersant for carbon nanotubes. RSC Adv..

[14] Hunter R.S., Van Mourik T. (2012). DNA base stacking: The stacked uracil/uracil and thymine/thymine minima. J. Comput. Chem..

[15] Zadorozhnaya A.A., Krylov A.I. (2010). Ionization-induced structural changes in uracil dimers and their spectroscopic signatures. J. Chem. Theory Comput..

[16] Bittner E.R. (2006). Lattice theory of ultrafast excitonic and charge-transfer dynamics in DNA. J. Chem. Phys..

[17] Kratochvíl M., Engkvist O., Šponer J., Jungwirth P., Hobza P. (1998). Uracil dimer: Potential energy and free energy surfaces. Ab initio beyond hartree-fock and empirical potential studies. J. Phys. Chem. A.

[18] Shieh Y.-T., Tu Y.-Y., Wang T.-L., Lin R.-H., Yang C.-H., Twu Y.-K. (2013). Apparent electrocatalytic activities of composites of self-doped polyaniline, chitosan, and carbon nanotubes. J. Electroanal. Chem..

[19] Xu D., Li B., Wei C., He Y.-B., Du H., Chu X., Qin X., Yang Q.-H., Kang F. (2014). Preparation and characterization of mno 2/acid-treated cnt nanocomposites for energy storage with zinc ions. Electrochim. Acta.

[20] Jang J., Bae J., Choi M., Yoon S.-H. (2005). Fabrication and characterization of polyaniline coated carbon nanofiber for supercapacitor. Carbon.

[21] Cheng C.-C., Chang F.-C., Ko F.-H., Yu F.-C., Lin Y.-T., Shieh Y.-T., Chen J.-K., Lee D.-J. (2015). Supramolecular polymeric micelles as high performance electrochemical materials. J. Mater. Chem. C.

[22] Prevoteau A., Soulié-Ziakovic C., Leibler L. (2012). Universally dispersible carbon nanotubes. J. Am. Chem. Soc..

[23] Kar P., Choudhury A. (2013). Carboxylic acid functionalized multi-walled carbon nanotube doped polyaniline for chloroform sensors. Sens. Actuators B Chem..

[24] Park S.-N., Park J.-C., Kim H.O., Song M.J., Suh H. (2002). Characterization of porous collagen/hyaluronic acid scaffold modified by 1-ethyl-3-(3-dimethylaminopropyl) carbodiimide cross-linking. Biomaterials.

[25] Seneewong-Na-Ayutthaya M., Pongprayoon T. (2015). Water-dispersible carbon nanotube prepared by non-destructive functionalization technique of admicellar polymerization. Diam. Relat. Mater..

[26] Zabihi O., Ahmadi M., Naebe M. (2015). One-pot synthesis of aminated multi-walled carbon nanotube using thiol-ene click chemistry for improvement of epoxy nanocomposites properties. RSC Adv..

[27] Wu T.-M., Lin Y.-W. (2006). Doped polyaniline/multi-walled carbon nanotube composites: Preparation, characterization and properties. Polymer.

[28] Mahanandia P., Schneider J.J., Engel M., Stühn B., Subramanyam S.V., Nanda K.K. (2011). Studies towards synthesis, evolution and alignment characteristics of dense, millimeter long multiwalled carbon nanotube arrays. Beilstein J. Nanotechnol..

[29] Sakurai S., Nishino H., Futaba D.N., Yasuda S., Yamada T., Maigne A., Matsuo Y., Nakamura E., Yumura M., Hata K. (2012). Role of subsurface diffusion and ostwald ripening in catalyst formation for single-walled carbon nanotube forest growth. J. Am. Chem. Soc..

[30] Jung Y., Head-Gordon M. (2006). A fast correlated electronic structure method for computing interaction energies of large van der waals complexes applied to the fullerene–porphyrin dimer. Phys. Chem. Chem. Phys..

[31] Huang K.W., Wu Y.R., Jeong K.U., Kuo S.W. (2013). From random coil polymers to helical structures induced by carbon nanotubes and supramolecular interactions. Macromol. Rapid Commun..

[32] Šponer J., Leszczynski J., Hobza P. (2001). Electronic properties, hydrogen bonding, stacking, and cation binding of DNA and rna bases. Biopolymers.

[33] Lizundia E., Oleaga A., Salazar A., Sarasua J. (2012). Nano-and microstructural effects on thermal properties of poly (l-lactide)/multi-wall carbon nanotube composites. Polymer.

[34] Li H., Li M., Guo W., Di H., Fang C., Yang B. (2014). Electrochemical application of titanium dioxide nanoparticle/gold nanoparticle/multiwalled carbon nanotube nanocomposites for nonenzymatic detection of ascorbic acid. J. Solid State Electrochem..

[35] De P.K., Neckers D.C. (2013). Polymer-quantum dot-carbon nanotube composites formation under visible light irradiation. J. Photochem. Photobiol. A Chem..

[36] Chen J., Li Y., Liu S., Wang G., Tian J., Jiang C., Zhu S., Wang R. (2013). Remarkable activity of pdir nanoparticles supported on the surface of carbon nanotubes pretreated via a sonochemical process for formic acid electro-oxidation. Appl. Surf. Sci..

[37] Ghasemi M., Daud W.R.W., Hassan S.H., Oh S.-E., Ismail M., Rahimnejad M., Jahim J.M. (2013). Nano-structured carbon as electrode material in microbial fuel cells: A comprehensive review. J. Alloy. Compd..

[38] Yu G., Liu X., Xing G., Chen S., Ng C.F., Wu X., Yeow E.K.L., Lew W.S., Sum T.C. (2013). Spatially-resolved ultrafast optical spectroscopy of polymer-grafted residues on cvd graphene. J. Phys. Chem. C.

[39] Mohamed M.G., Hsu K.-C., Kuo S.-W. (2015). Bifunctional polybenzoxazine nanocomposites containing photo-crosslinkable coumarin units and pyrene units capable of dispersing single-walled carbon nanotubes. Polym. Chem..

[40] Yan H., Li D., Zhang Y., Yang Y., Wei Z. (2014). Rational design of ternary-phase polymer solar cells by controlling polymer phase separation. J. Phys. Chem. C.

[41] Hu T., Han L., Xiao M., Bao X., Wang T., Sun M., Yang R. (2014). Enhancement of photovoltaic performance by increasing conjugation of the acceptor unit in benzodithiophene and quinoxaline copolymers. J. Mater. Chem. C.

[42] Gupta S.K., Sahu M., Krishnan K., Saxena M., Natarajan V., Godbole S. (2013). Bluish white emitting sr 2 ceo 4 and red emitting sr 2 ceo 4: Eu 3+ nanoparticles: Optimization of synthesis parameters, characterization, energy transfer and photoluminescence. J. Mater. Chem. C.

[43] Ogihara H., Xie J., Saji T. (2014). Spraying carbon nanotube dispersions to prepare superhydrophobic films. J. Mater. Sci..

