# Supplementary material for: Bio-Inspired Supramolecular Chemistry Provides Highly Concentrated Dispersions of Carbon Nanotubes in Polythiophene
Source: Materials (Basel). 2016 Jun 2;9(6):438. doi: 10.3390/ma9060438 (PMC5456781; doi:10.3390/ma9060438)
Supplement: Supplementary file 1 [file materials-09-00438-s001.pdf]

Supplementary Materials: Bio-Inspired Supramolecular Chemistry Provides Highly Concentrated Dispersions of Carbon Nanotubes in Polythiophene

Yen-Ting Lin, Ranjodh Singh, Shiao-Wei Kuo and Fu-Hsiang Ko

Synthesis of 3-(Adeninehexyl Thiophene)

We dissolved 3-bromothiopene (*a*, 30 g, 0.18 mol) in 250 mL of n-hexane and decreased the temperature to −40 °C. After injection of n-BuLi, the solution was stirred for 10 min, and then, THF was added slowly through a syringe (15~20 mL) until an insoluble white precipitate of 3-lithiothiophene salt was formed. We ramped the temperature from −40 °C to −10 °C and stirred for 1 h. Dried THF (5~10 mL) and 1,6-dibromohexane (*b*, 110 mL, 0.72 mol) were added in the mixture and then stirred for 2 h at room temperature. When the reaction was complete, we used ether and water for extraction. After rotary evaporation of the organic phase, we gained a colorless and oily precursor (*c*, 27.39 g, yield 61%) under low-pressure (90~100 °C, 0.05 mmHg) distillation. We dissolved the measured precursor (*c*, 2.79 g, 20.0 mmol), calcium carbonate (5.58 g, 40.0 mmol)and adenine (5.40 g, 40 mmol) in dried DMF (200 mL) and stirred the mixture under 60 °C for 24 h. When the reaction was complete, we cooled it to room temperature, filtered out insoluble solids and removed the DMF by low-pressure distillation. Finally, we re-crystallized the mixture in toluene to obtain 3-adeninehexyl thiophene monomer (*d*, 5 g, yield 45%). The monomer structure was confirmed by ^{1}H NMR (300 MHz, $CDCl_3$) δ = 1.34 (m, 4H), 1.65 (br, 2H), 2.60 (m, 2H), 3.68 (t, 2H), 5.65 (d, 1H), 6.89 (d, 1H), 7.08 (d, 1H), 7.21 (d, 1H), and 8.80 (s, 1H) ppm and ^{13}C NMR (75 MHz, $CDCl_3$): δ = 26.45, 28.96, 29.19, 30.30, 30.49, 49.06, 102.34, 120.17, 125.45, 128.40, 142.96, 144.65, 151.15, and 164.18 ppm.

Synthesis of Poly(3-Adeninehexyl Thiophene) (PAT)

We placed $FeCl_3$ (4 g, 137 mol) in a 250 mL double-neck bottom with dehumidified air under Ar atmosphere. We used a small amount of dried chloroform to dissolve the $FeCl_3$ in a cooling bath and diluted 3-adeninehexyl thiophene (*d*, 1.73 g, 5.74 mmol) in 20 mL of dried chloroform. We slowly injected 3-adeninehexyl thiophene dilution in $FeCl_3$ solution under a cooling bath and allowed the reaction to proceed for several days to ensure that polymerization was complete. We removed the chloroform by low-pressure distillation and added methanol and a small amount of hydrazine to wash the crude product. We further purified the mixture by Soxhlet Extraction and removed methanol to obtain poly(3-adeninehexyl thiophene) (PAT), which was confirmed by ^{1}H NMR (400 MHz, DMSO-d_6): δ = 1.20 (m, 4H), 1.53 (m, 2H), 1.71 (m, 2H), 2.65 (t, 2H), 4.05 (t, 2H), 7.07 (s, 1H), 7.20 (s, 2H), 8.08 (s, 2H) (Figure S2). IR (KBr): 3250 cm^{-1}, 2835 cm^{-1}, 1615 cm^{-1}, 520 cm^{-1}, and 710 cm^{-1} (Figure 1) and MS (EI), m/z [M^{+}]: 2840, calculated: 2840.68.

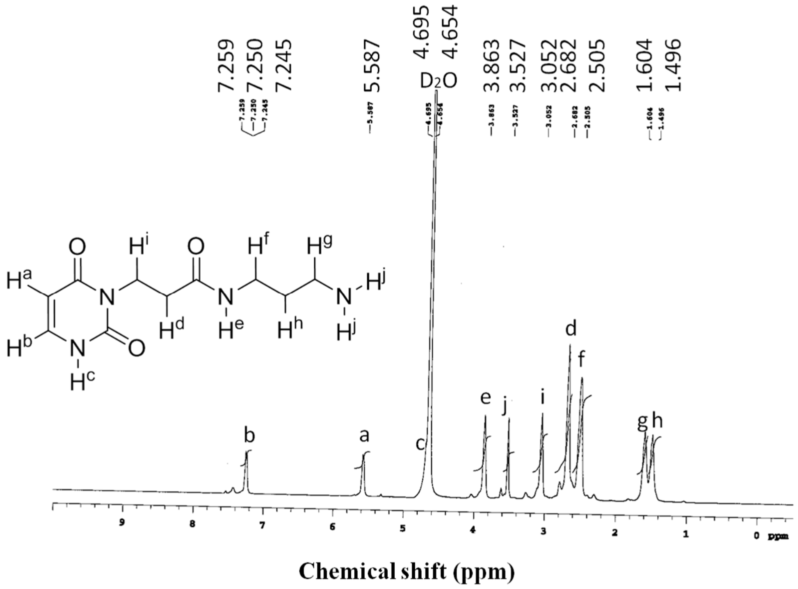

Figure S1. ^{1}H NMR result for U derivative.

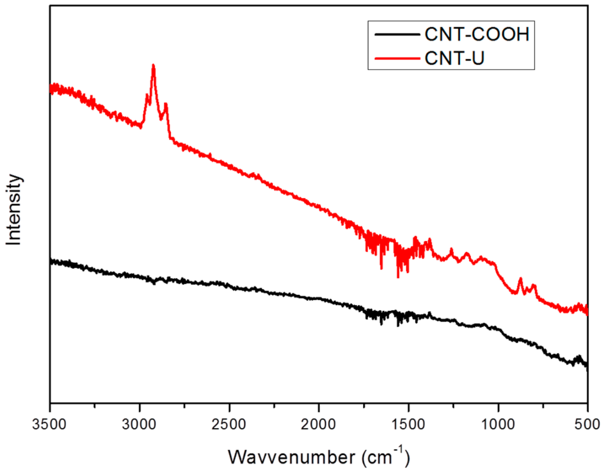

Figure S2. FT-IR (KBr pellet) result for monitoring modification of CNT-COOH (black) and grafted CNT-U (red).

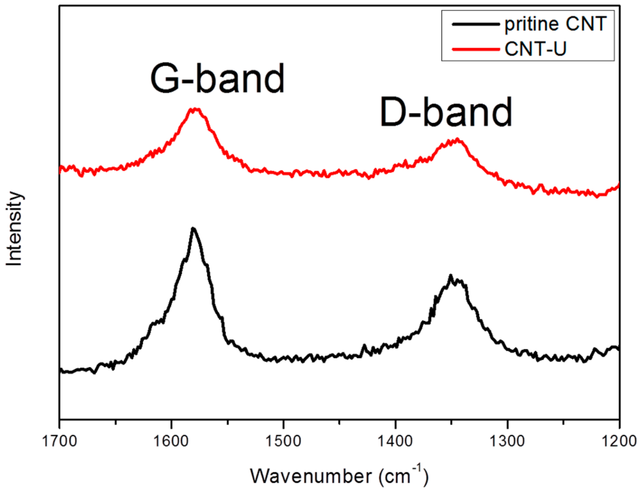

Figure S3. Raman result for monitoring modification of non-modified CNT-COOH (black) and grafted CNT-U (red).

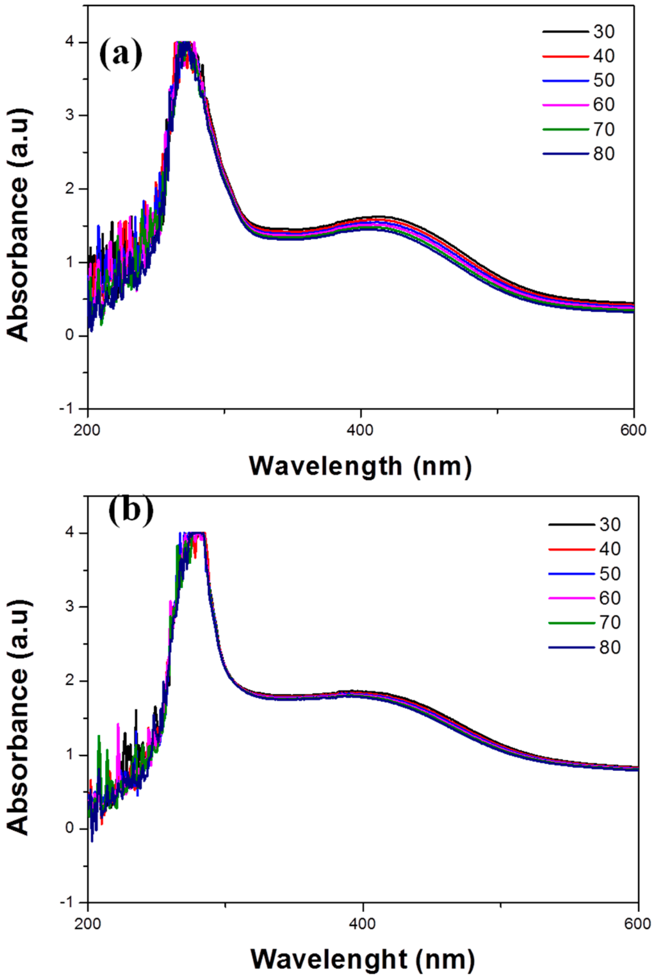

Figure S4. Stability of (**a**) commercial MWCNT-COOH and (**b**) self-made MWCNT-COOH by temperature oscillation study (30–80 °C) by UV-vis spectra.

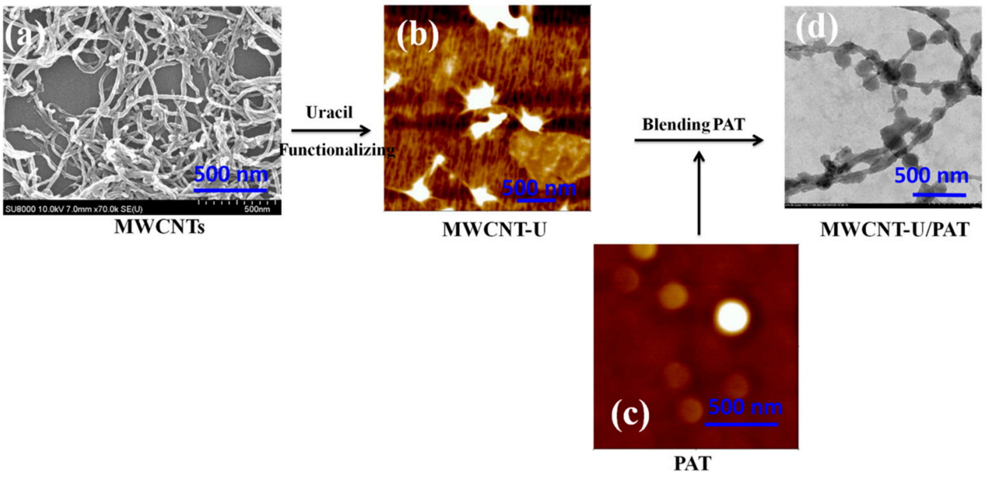

Figure S5. The structure for (**a**) CNT-COOH (SEM); (**b**)CNT-U (AFM); (**c**) PAT (AFM) and (**d**) CNT-U/PAT (TEM).

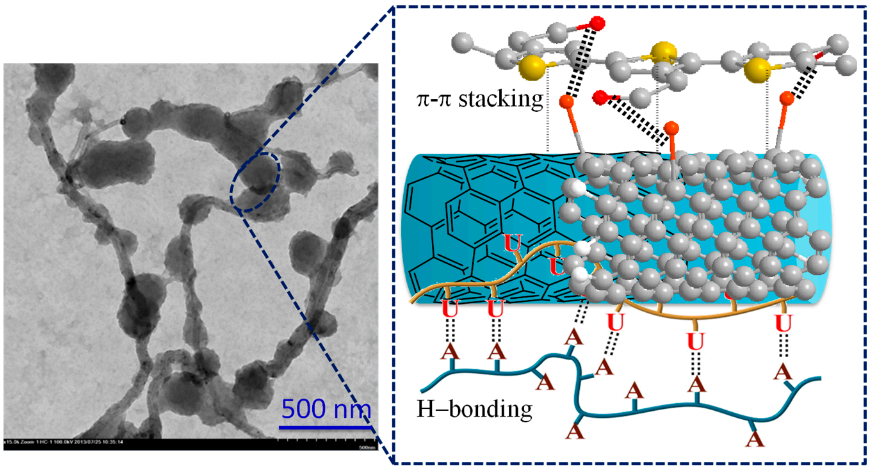

Figure S6. Shows the π-stacking interactions between the surface of CNT-U and the thiophene rings of PAT; the U···A multiple hydrogen bond interaction enhanced the dispersion of CNT-U in the PAT solution.
